# Implications of Diabetes-Induced Altered Metabolites on Retinal Neurodegeneration

**DOI:** 10.3389/fnins.2022.938029

**Published:** 2022-07-13

**Authors:** Dalia I. Aldosari, Ajamaluddin Malik, Abdullah S. Alhomida, Mohammad S. Ola

**Affiliations:** Department of Biochemistry, College of Science, King Saud University, Riyadh, Saudi Arabia

**Keywords:** metabolites, glucose, lipids, amino acids, neurodegeneration, retina, oxidative stress

## Abstract

Diabetic retinopathy (DR) is one of the major complications of diabetic eye diseases, causing vision loss and blindness worldwide. The concept of diabetic retinopathy has evolved from microvascular disease into more complex neurovascular disorders. Early in the disease progression of diabetes, the neuronal and glial cells are compromised before any microvascular abnormalities clinically detected by the ophthalmoscopic examination. This implies understanding the pathophysiological mechanisms at the early stage of disease progression especially due to diabetes-induced metabolic alterations to damage the neural retina so that early intervention and treatments options can be identified to prevent and inhibit the progression of DR. Hyperglycemia has been widely considered the major contributor to the progression of the retinal damage, even though tight control of glucose does not seem to have a bigger effect on the incidence or progression of retinal damage that leads to DR. Emerging evidence suggests that besides diabetes-induced hyperglycemia, dyslipidemia and amino acid defects might be a major contributor to the progression of early neurovascular retinal damage. In this review, we have discussed recent advances in the alterations of key metabolites of carbohydrate, lipid, and amino acids and their implications for neurovascular damage in DR.

## Introduction

Diabetic retinopathy (DR) is one of the most common diabetic eye diseases, which causes preventable vision impairment and blindness worldwide. According to a recent report, approximately 537 million people are diabetic worldwide, over one-third have signs of DR, and nearly 1 in 10 have vision-threatening levels of DR, including severe non-proliferative DR pro-liferative DR (PDR), and diabetic macular edema (DME) (Federation, 2021; Teo et al., 2021). These estimates are expected to rise further due to the increasing prevalence of diabetes, aging global population, lifestyle changes, and growing lifespan of people living with diabetes (Teo et al., 2021). Current treatments such as laser surgery and intraocular injection of anti-VEGF agents only target the advanced stage of DR, including ME and PDR. These treatments are temporarily effective and usually do not promote tissue repair and sometimes can impair vision; in some patients, the retinopathy continues to progress (Narayanan et al., 2013). Therefore, a better understanding of the pathogenesis of DR in the early stages of the disease would permit the development of more efficient strategies against the progression of DR.

Over the last two decades, the concept of diabetic retinopathy has evolved from microvascular disease into more complex neurovascular diseases in which neurodegeneration plays a significant role in the very early stages of the disease. Retinal neurodegeneration has been found to trigger vascular injury which leads to diabetic retinopathy at the later stages of diabetes (Feng et al., 2009; Moran et al., 2016). However, the exact reasons for neuro-vascular cell damage early in the diabetic retina are not well known.

**Figure 1:**
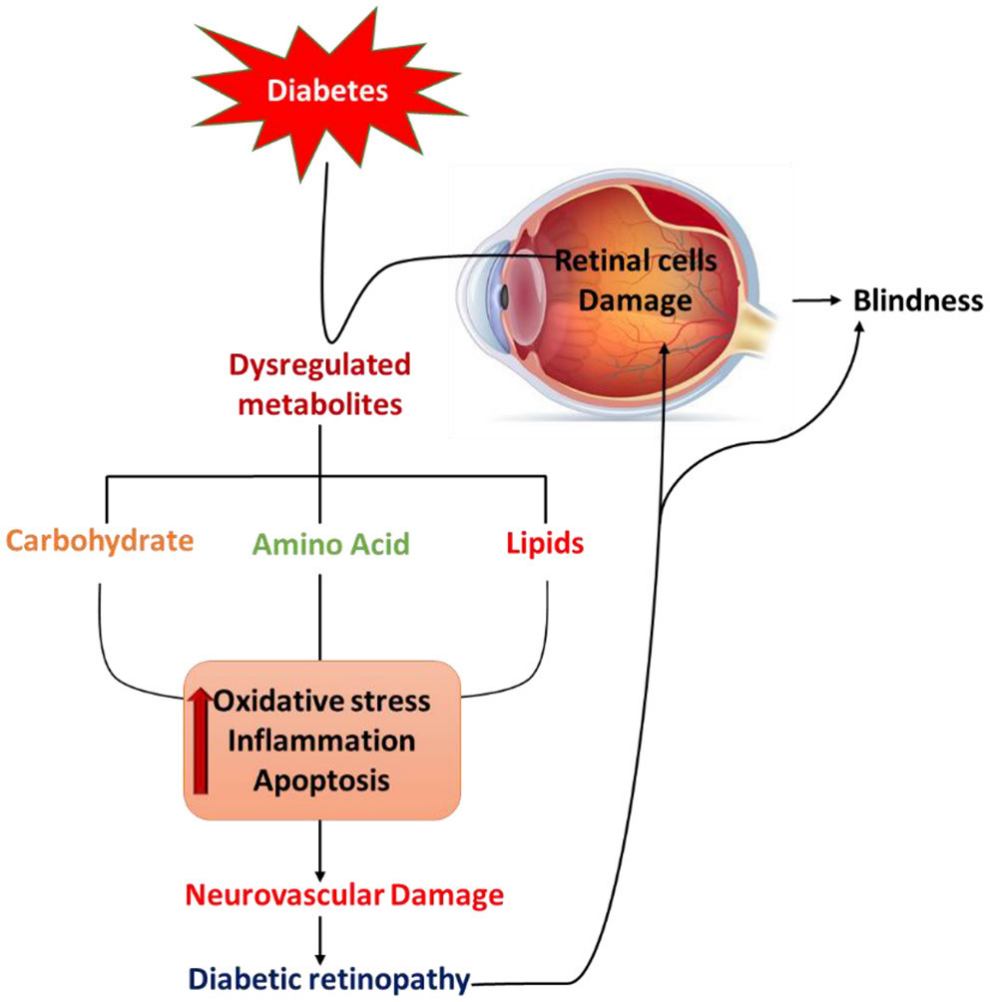
Schematic diagram summarizes diabetes-induced pathological defects in the carbohydrate, lipid, and amino acid metabolism, leading to increases in oxidative stress, activation of apoptosis, and inflammation that may contribute to retinal cell damage and diabetic retinopathy.

Retina being highly metabolic active sensory tissue demands an increased amount of energy metabolites for maintaining energy homeostasis and neurotransmitter regulation for normal vision (Kern, 2014; Liu and Prokosch, 2021). However, diabetes disrupts several of those energy metabolites in the retinas of diabetic patients and rodents both systemically and locally. Diabetes-induced hyperglycemia, dyslipidemia, and defects in amino acid metabolism are crucial factors in disturbing the energy metabolism. Numerous studies, suggest that the pathogenesis of DR is potentially related to elevated levels of numerous metabolites of glucose, lipids, and amino acids (Ola and Alhomida, 2014; Chou et al., 2020; Yumnamcha et al., 2020) as depicted in Figure 1. Those metabolites may include advanced glycation end products, polyols, hexosamines, excitatory amino acids; glutamate, homocysteine, branched-chain amino acids, cholesterol, polyunsaturated fatty acids, and sphingolipids; whose dysregulated levels may cause neurodegeneration in the diabetic retina by increasing oxidative stress and decreasing neurotrophic support, leading to the progression of DR. In this review, we have highlighted the role of potential metabolites which play a significant role in the pathophysiology of retinal damage in diabetes and more specifically in the neuronal cells damage in retinas and that have been correlated with DR progression. This article would provide a better understanding and guidance to researchers in fostering further research and contributing to the understanding of metabolic stress in DR.

### Dysregulated Glucose Metabolism

**Figure 2:**
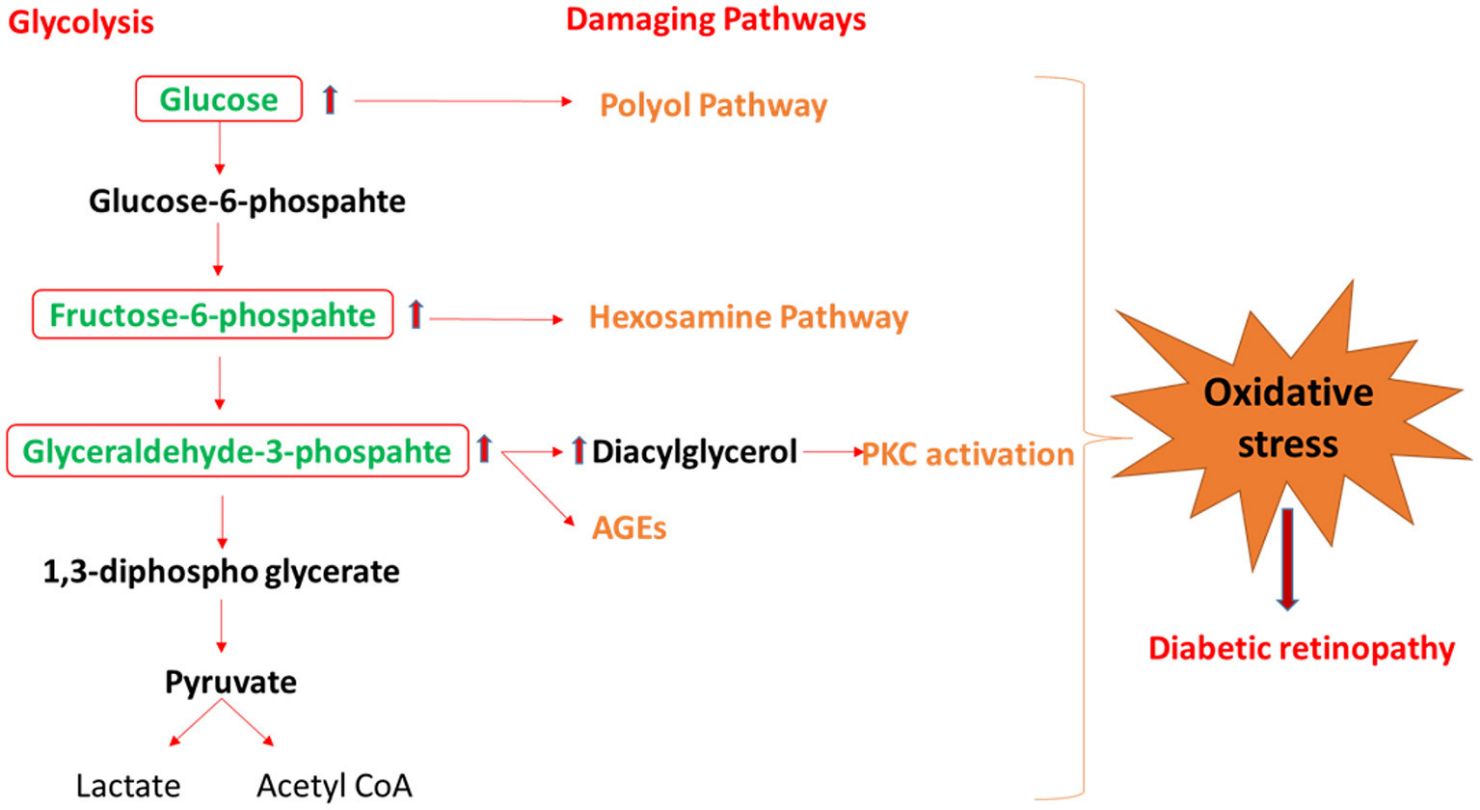
The schematic diagram shows the mechanism by which glycolysis intermediates overload under diabetic condition and activates the damaging pathway of polyol, hexosamine, PKC, and AGEs, that lead to diabetic retinopathy. PKC, protein kinase C; AGEs, advance glycation end-products.

The retina is one of the highest energy-demanding tissues of the human body, and it relies almost exclusively on glucose as its energy source. Therefore, maintenance of euglycemia is critical for normal vision and regulating energy homeostasis in the retina. Glucose homeostasis is tightly controlled by the interplay between glycolysis, Krebs cycle, and oxidative phosphorylation. Many glucose intermediates are produced through the glycolytic pathway, including glucose-6-phosphate, Fructose-6-phosphate, dihydroxyacetone phosphate (D), and glyceraldehyde 3-phosphate, 3-phosphoglycerate, and phosphoenolpyruvate. Under physiological conditions, the retina, similar to the brain depends on the glycolytic pathway to obtain a significant amount of energy and also maintain a steady-state concentration of glycolytic intermediates (Chinchore et al., 2017). However, increased flux through glycolysis, which is commonly seen under diabetic conditions, results in glycolytic overload, which leads to an abnormal increase in the concentrations of glycolytic intermediates that can be shunted into different damaging pathways such as polyol, hexosamine, protein kinase C (PKC) pathways, and advanced glycation end products (AGEs) as shown in Figure 2 (Yumnamcha et al., 2020). The activation of these damaging pathways leads to an increase in oxidative stress either by the generation of reactive oxygen species (ROS) or by decreasing the level of antioxidants, as summarized below.

### Polyol Formation

Under physiological conditions, glucose is phosphorylated by an enzyme called hexokinase; however, when there is excess glucose uptake, this enzyme becomes saturated, and any excess glucose is shunted into the polyol pathway. The polyol pathway involves the conversion of glucose into sorbitol, and the reaction is catalyzed by aldose reductase. Unlike under diabetic conditions, the high activation of the polyol pathway could enhance oxidative stress because aldose reductase requires NADPH as a cofactor, thereby reducing the NADPH level, which is a necessary cofactor for regenerating the intracellular antioxidants. In addition, NADPH is a cofactor for glutathione reductase, which converts oxidized glutathione (GSSG) to the critical cellular reductant, reduced glutathione (GSH). Furthermore, NADPH is required for many anabolic pathways such as nitric oxide and fatty acid synthesis, however, NADPH consumption in the aldose reductase pathway under diabetic conditions causes a harmful effect on the biosynthesis process of these molecules in the retina (Thakur et al., 2021).

Polyol pathway activation has been found in various retinal cells, such as retinal ganglion cells (RGC), and Müller cells under hyperglycemic conditions and in nerve fibers in diabetic patients (Dagher et al., 2004). Many studies have proved that sorbitol is increased in the retina of diabetic animals and diabetic patients (Dagher et al., 2004; Lorenzi, 2007; Ola et al., 2012). The increased levels of sorbitol through the polyol pathway can alter the osmotic pressure of the membrane and cause osmotic stress, which causes electrolyte imbalance, hydration and membrane damage (D'Andrea et al., 2020; Thakur et al., 2021). Additionally, polyol pathway activation in the retina of diabetic rats shows increased lipid peroxidation products and depletion of antioxidant enzymes, suggesting that retinal cells and more specifically neuronal cells might be more prone to oxidative stress induced by the polyol pathway early in diabetes (Ola et al., 2018). Furthermore, recent studies showed that the aldose reductase inhibitors helped to slow DR progression (D'Andrea et al., 2020; Thakur et al., 2021).

## Activation of Hexosamine Pathway

The hexosamine biosynthesis pathway is activated as an alternative to the glycolysis pathway by shunting hyperglycemia-induced overproduction of fructose-6-phosphate to glucosamine-6-phosphate by fructose-6- phosphate amidotransferase (GFAT), and further to uridine-5-diphosphate (UDP)-N-acetylglucosamine (UDP-GlcNAc), which is essential to the formation of O-linked glycoproteins (Du et al., 2000). Alternative O-linked glycoprotein can have diverse effects on cell function and survival by disrupting gene expression of several proteins in the retina, especially in neurovascular cells (Fülöp et al., 2007). Under the diabetic condition, activation of the hexosamine pathway contributes to the pathogenesis of DR by blocking the neuroprotective effect of the insulin/Akt signaling pathway that leads to increases retinal neuronal cell death (Kim et al., 2016). It also promotes retinal ganglion cell death in the retina of diabetic rodents by increasing O-GlcNAcylation of the NF-κB p65 subunit and its activation [reviewed in Yumnamcha et al. (2020)]. In addition, recent research found increased production of UDP-N-acetyl glucosamine under diabetic conditions. This product of the hexosamine pathway competes with phosphorylation at post-translation modification sites on transcription factors and disrupts the regulation of inflammatory responses that generally occurs through transcription growth factor-β (Yumnamcha et al., 2020). Thus, the activation of the hexosamine pathway under diabetic conditions leads to neurodegeneration in the retina, specifically through the activation of pro-inflammatory transcription factors.

## Activation of Protein Kinase C Pathway

Under hyperglycemic conditions, the high intracellular glucose concentration induces a *de novo* pathway of diacylglycerol (DAG) synthesis from glucose to glycerol 3-phosphate, which forms glycerol phosphate (Geraldes and King, 2010; Giacco and Brownlee, 2010). DAG activates different isoforms of protein kinase C (PKC) within the cell, which play an essential role in the pathogenesis of DR by several mechanisms. First, activation of PKC by DAG causes retinal vascular dysfunctions and pericyte losses which is the principal hallmark of the pathogenesis of microvascular damage in DR. Second, the accumulation of DAG under diabetic condition activates PKC-β, which increase the expression of endothelium-derived vasoactive factors such as endothelin-1(ET-1), vascular permeability, and angiogenesis as well as an increase in vascular endothelial growth factor (VEGF) that affect the blood flow in the retina. Third, the increased production of DAG as a result of hyperglycemia under diabetic conditions also activate PKC-δ, which induces pericyte loss through; increasing oxidative stress by activating nuclear factor-B (NF-κB) and upregulating the expression of a protein tyrosine phosphatase, to weaken the critical survival signaling pathway of platelet-derived growth factor (Yumnamcha et al., 2020). Therefore, PKC activation might be partly responsible for some of the pathologies in DR.

### Advanced Glycation End Products

Diabetes-induced high intracellular glucose concentration causes accumulation of the production of advanced glycation end products (AGEs). Increased AGEs formation has been found in retinal blood vessels of diabetic patients and animals and in human serum and vitreous of diabetic patients, which correlate with the severity of retinopathy (Ola et al., 2012). AGEs exert their cellular effects on cells by activating their receptor, RAGE, which contributes to activating the NADPH oxidase system and increasing the production of intracellular ROS (Rains and Jain, 2011). The ROS produced, in turn, increases VEGF, monocyte chemoattractant protein- 1 (MCP-1), and intercellular adhesion molecule-1 (ICAM-1) expression in microvascular endothelial cells, thus leading to leukostasis and breakdown of BRB (Ola et al., 2012). AGEs also induce apoptosis and inflammation by activating NF-κB, with a simultaneous increase in the ratio of Bcl-2/Bax, and activity of caspase-3, a key enzyme in the execution of apoptosis of pericytes (Ola and Nawaz, 2012).

Although hyperglycemia is known to cause a higher risk for the development and progression of DR, however, tight control of glucose does seem to improve the incidence or progression of DR. Even aggressive intervention to improve glycemia may raise the possible increased risk of the retinal damage (Ipp and Kumar, 2021). Our studies including a few others suggest that hyperglycemia per se might not cause oxidative stress in the diabetic retina and cultured retinal cells (Ola, 2021). Therefore, it is imperative that dysregulated metabolites of lipids and amino acids, other than high glucose might be other major metabolic factors that might implicate in the pathophysiology of early retinal damage in DR.

### Lipid Metabolism in Diabetic Retinopathy

The retina is a heavily energy-consuming tissue used for several processes, including phototransduction, visual pigment recycling, and synaptic activity. However, Cohen and Noell (1960), reported that almost 65% of the CO_2_ produced from the TCA cycle by the retinas is not derived from glucose, which implies that the oxidation of non-carbohydrate carbons is also used to meet the retinal high energy demand (Joyal et al., 2018; Millman et al., 2019). Furthermore, several studies recently found that lipid oxidation acts as another energy source for the retina (Joyal et al., 2016). In addition, for decades numerous research has highlighted the significant role of lipids in retinal function by transferring many signaling through membrane proteins. Retinal lipids also play an essential role in the visual cycle and converting all-trans-retinal to 11-cis retinal (Zemski Berry et al., 2014). Additionally, very long-chain polyunsaturated fatty acids (VLC-PUFA), such as docosahexaenoic acid (DHA), are disproportionately abundant in the retina compared with other tissues (Zemski Berry et al., 2014). The levels of DHA affect the fluidity, flexibility, and thickness of any membrane. Furthermore, the large amount of DHA in the retina permits efficient conformational changes in rhodopsin (Zemski Berry et al., 2014). It is now well known that modifications in fatty acid metabolism can affect various retinal functions and contribute to retinal diseases that lead to visual loss.

**Figure 3:**
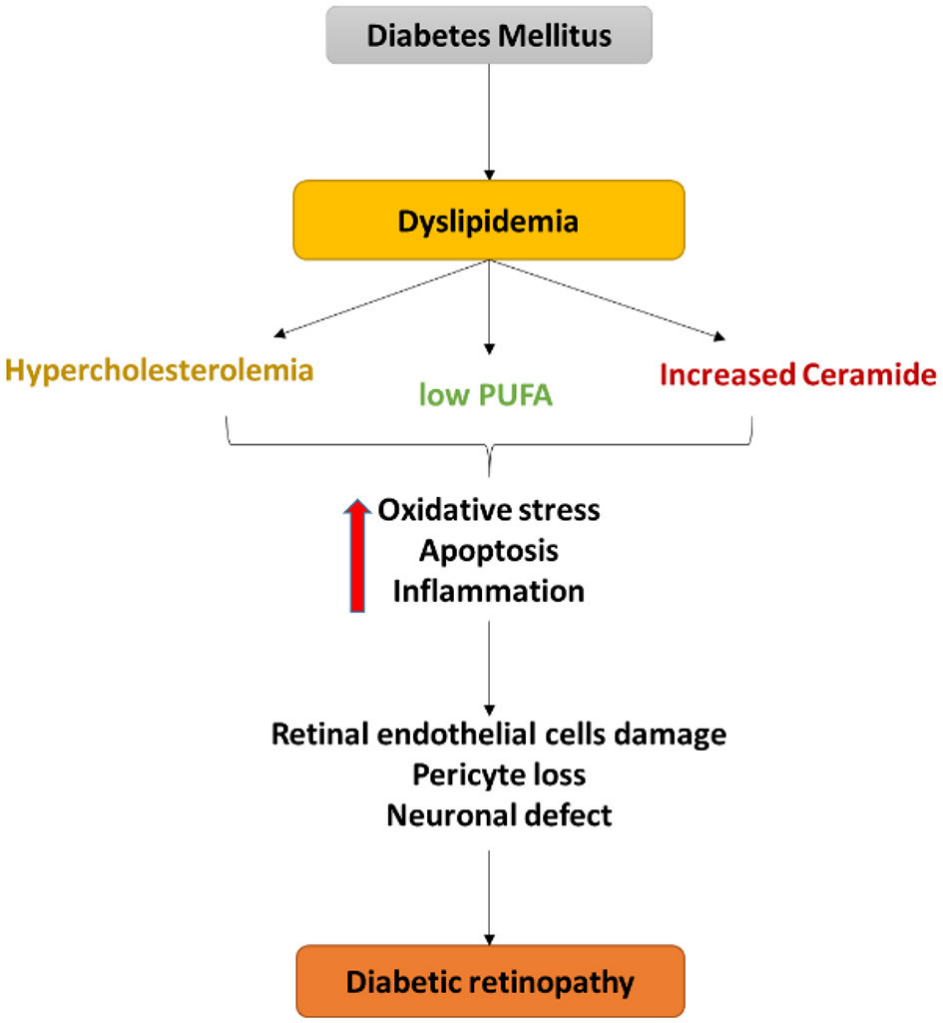
Schematic diagram of the central role of dyslipidemia in endothelial cell damage, pericyte loss as well as a neuronal defect in diabetic retinopathy. Dyslipidemia in diabetes induces oxidative stress, apoptosis, and inflammation that contribute to the retinal neurovascular damage in DR.

Abnormalities in retinal lipid metabolism occur early during diabetes. Diabetic dyslipidemia produces a deleterious effect on the retina. Dyslipidemia is characterized by abnormal circulating of triglycerides, high-density lipoproteins (HDL), low-density lipoproteins (LDL), cholesterol, and polyunsaturated fatty acids. Dyslipidemia in DR is summarized in a series of reviews as depicted in Figure 3 (Busik et al., 2012; Hammer and Busik, 2017; Fu et al., 2019; Chou et al., 2020; Busik, 2021), and here we have focused on hypercholesterolemia, PUFA, and sphingolipids in DR.

### Hypercholesterolemia

Cholesterol levels in the retina are affected by several factors, including systemic delivery, local biosynthesis, and cholesterol elimination (Hammer and Busik, 2017; Busik, 2021). The inner blood-retinal barrier (inner BRB) is created by complex tight junctions of retinal capillary endothelial cells, which, when intact, are impervious to cholesterol. However, the breakdown of inner BRB in DR could lead to the non-specific entry of lipoprotein particles into the retina, increasing retinal cholesterol levels (Busik, 2021). The outer BRB, formed by the retinal pigmented epithelium (RPE) cells, which contain the Low-Density Lipoprotein Receptor (LDLR), allows for cholesterol transport into the retina obtained from the diet or produced by the liver (Hammer and Busik, 2017). After uptake by the RPE, cholesterol is exported by ABCA1- and ABCG1-transporters either back to the liver in a multi-step process by reverse cholesterol transport or to the neural retina. In addition to exporting cholesterol, both RPE and neural retina metabolize cholesterol to more soluble oxysterols that rapidly diffuse to the systemic circulation by cytochrome P450s (CYPs) enzymes 27A1, and 46A1, key enzymes in cholesterol elimination (Hammer and Busik, 2017; Busik, 2021). Obstructing control of cholesterol metabolism pathways can cause retinal cholesterol accumulation, negatively affecting normal retinal function.

Several studies demonstrated that increased cholesterol levels pass through BRB in the diabetic retina compared to the non-diabetic retina (Fliesler et al., 1993; Hammer and Busik, 2017). In addition, abnormal cholesterol elimination in the diabetic retina leads to hypercholesterolemia increasing non-enzymatic oxidation and glycation (Hammer and Busik, 2017). Oxidized and glycated LDL induced retinal pericyte loss and oxidized LDL immunocomplexes in diabetic retinopathy (Fu et al., 2014). In addition, numerous studies have described the neurotoxic effects of hypercholesterolemia by induced amyloid-beta peptide accumulation (Rao et al., 2021). Elevated levels of Ab peptide in the retina lead to increased production of ROS which has deleterious effects on retinal ganglion cells (Rao et al., 2021). Furthermore, hypercholesterolemia produces several oxidized byproducts such as 7-ketocholesterol (7-kCh) that result in retinal damage. 7-kCh is a potent pro-apoptotic and inflammatory agent shown to activate caspases, p38, MAP/ERK, and AKT-PKCz-NF-kB (Hammer and Busik, 2017; Rao et al., 2021). Activation of the MAP/ERK pathway increases oxidative stress, inflammation, and apoptosis in retinal neurovascular cells (Rao et al., 2021). In addition, activation of the NF-kB pathway by 7kCh facilitates the expression of many cytokines that result in an inflammatory response (Rao et al., 2021). Furthermore, Yang et al. (2019), found that injecting rat retina with 7KCh mainly deposited in the retinal pigment epithelial (RPE) cells and induced marked photoreceptor apoptosis (Yang et al., 2019). Increasing evidence has suggested a positive correlation between hypercholesterolemia and the risk and the severity of hard exudate among diabetic patients (Chou et al., 2020). In addition, the diabetic patients with higher circulating LDL-C were also more inclined to suffer from diabetic retinal hard exudate. Furthermore, lipid-lowering dietary therapy studies demonstrated regression of retinal hard exudates and beneficial effects toward amelioration of the progression of DR (Busik et al., 2012).

### Polyunsaturated Fatty Acid (PUFA)

Both systemic and retinal-specific fatty acid profiles are affected by diabetes, especially DHA, the most abundant PUFA in the retina. Several animal and cell culture studies demonstrated that the DHA has anti-inflammatory by inhibiting cytokine-induced NF-κB activation, nuclear translocation, adhesion molecule expression, and anti-apoptotic effects in the retina and retinal cells (Busik et al., 2012). In addition, DHA contributes to cholesterol homeostasis in the retina by incorporation into phospholipids of caveolar membrane microdomains and displacement of cholesterol from these microdomains as essential mechanisms of the anti-inflammatory effects of DHA in the human retinal endothelial cells (HREC) (Busik et al., 2012). However, diabetes has been found to reduce levels of DHA in both the diabetic retina and plasma, with a concomitant increase in pro-inflammatory omega-6 PUFA that was found to contribute to the development of DR (Tikhonenko et al., 2013). *In vivo* experiments revealed that a diet rich with a DHA at the recommended levels is protective against capillary loss in the animal model of diabetic retinopathy. In both type 1 and type 2 diabetic animal models, a DHA-rich diet entirely prevented retinal vascular pathology by inhibiting acid sphingomyelinase (ASM) in the retina and endothelial progenitor cells (EPCs), leading to simultaneous suppression of retinal inflammation and correction of EPC number and function (Tikhonenko et al., 2013). In addition, several studies demonstrated that increased dietary intake of DHA prevents vascular pathology through inhibition of ASM in the retina, leading to an inhibition of retinal endothelial cell activation by inflammatory cytokines and reducing pathological retinal angiogenesis (Busik et al., 2012).

### Sphingolipids

Diabetes has also been shown to affect sphingolipid metabolism (Fox et al., 2006; Hammer and Busik, 2017). Sphingolipids are a class of biologically active lipids that have essential roles in regulating tissue development, cell death, inflammation, adhesion, and migration (Hammer and Busik, 2017). One of the primary intermediates involved in sphingolipid metabolism is known as ceramide. This intermediate is a potent messenger in stress signaling and pro-apoptotic sphingolipid that accumulates in endothelial and immune cells (Hammer and Busik, 2017). Ceramide is mainly synthesized through the breakdown of sphingomyelin by sphingomyelinases. There are three types of sphingomyelinases classified depending on the pH optimum, acid, neutral, and alkaline. The alkaline form is only expressed in the gut, while acid and neutral sphingomyelinases are ubiquitously expressed in most tissues (Busik et al., 2012). Both acid and neutral sphingomyelinases are expressed in the retina (Opreanu et al., 2011). Evidence suggests that the ASM is activated by diabetes (Busik et al., 2012; Tikhonenko et al., 2013; Hammer and Busik, 2017). Activation of ASM leads to higher sphingomyelin-ceramide conversion causing retinal endothelial damage in the diabetic retina, macrophage, and microglial activation, and circulating angiogenic cell dysfunction (Hammer and Busik, 2017). In addition, inhibition of ASM in the retina has been shown to prevent diabetes-induced vascular degeneration and cytokine-induced pro-inflammatory changes (Busik et al., 2012).

### Amino Acid Metabolism in the DR

**Figure 4:**
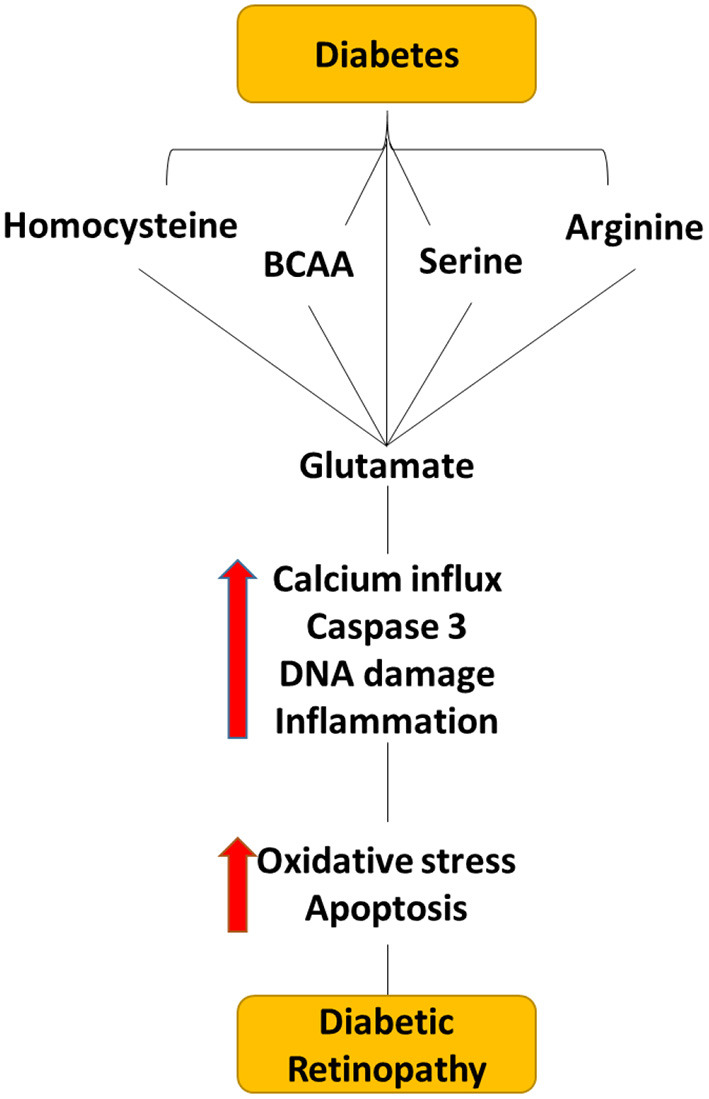
Flow diagram depicting the effect of dysregulated level of amino acid within the diabetic retina. Dysregulated levels of homocysteine, serine, BCAA, arginine, and glutamate cause elevating the glutamate levels in the extracellular space within the retina, which can cause neuro-vascular damage in diabetic retinopathy.

Amino acids (AAs) and their metabolites play a critical role in retinal health and function (Pow, 2001). They are not only used for protein synthesis, but some amino acids act as a significant neurotransmitter in the retina (Pow, 2001; Bui et al., 2009). In addition, the central nervous system, including the retina, requires amino acid and nitrogen balance, however, any changes in the AA metabolism can cause neurological dysfunction and irreversible damage. Many studies have reported altered amino acid levels in serum and retina in DR patients and DR models of rodents (Diederen et al., 2006; Narayanan et al., 2013; Ola et al., 2013). Altered glutamate, glutamine, arginine, tryptophan, BCAA, and homocysteine metabolism have been found to have a greater impact on DR as depicted in Figure 4 (Ola et al., 2013, 2019). Metabolic abnormalities of these amino acids lead to an increase in oxidative stress, and inflammation and induce apoptosis, thus inducing neuroretina damage in diabetes as discussed below.

## Excitatory Amino Acids (Glutamate)

Glutamate is a major neurotransmitter in the retina and acts as a precursor for gamma-aminobutyric acid (GABA), an inhibitory neurotransmitter, and glutathione, an endogenously antioxidant; therefore, any changes in glutamate metabolism in the retina have a more significant effect on neuroretinal functioning (Pow, 2001). Photoreceptors, bipolar, and ganglion cells in the retina release glutamate, during light stimulation under the regular state and activate the N-methyl-D-aspartate receptor (NMDA). This Glu receptor is expressed in most cells of neuro-retinal cells (Shen et al., 2006). The activation of NMDA leads to depolarizing of the neuronal cells and increases the influx of calcium and sodium ions into the cell (Shen et al., 2006). However, prolonged exposure of neurons to glutamate leads to cell death by elevating the intracellular Ca^2+^ that subsequently generates free radicals and induces apoptosis in the neuronal cells (Ola et al., 2013). Glu receptors' activation control is achieved by Glu clearance from the synaptic cleft by glutamate transporter proteins expressed on both neurons and Müller cells (Ishikawa, 2013). Within neurons, glutamate can be recycled, while in Müller cells, glutamate is inactivated by conversion to less toxic metabolites such as glutamine and alpha-ketoglutarate through the glial L-glutamate/L-aspartate transporter (GLAST), which is expressed in the Müller cells, and glutamine synthetase (GS), which is described in the cytoplasm of Müller cells (Bringmann et al., 2013). All of this maintain the requisite glutamate homeostasis in the retina after excitation. However, many lines of evidence demonstrate a defect controlling systems of Glu levels in the diabetic retina (Gowda et al., 2011; Ishikawa, 2013). Considerable evidence indicates an increase in Glu levels in the vitreous and retina of diabetic patients with PDR and experimental animal models of diabetes (Diederen et al., 2006; Ishikawa, 2013; Ola et al., 2019). The elevated levels of Glu in the diabetic vitreoretinal are thought to be mediated by the dysfunction of glutamate transporters on retinal Müller cells and possibly, due to the decreased expression of glutamine synthase (Li and Puro, 2002; Ishikawa, 2013). Lau et al. (2013), reported a significant decrease in the expression of glutamate transporter SLC1A3 gene encoding GLAST protein, leading to the decreased removal of glutamate from the synaptic space due to impairment of the glutamate transporter function of Muller cells. Besides the elevated level of Glu in the DR, it also has been observed the high expression of NMDA in experimental diabetes (Smith, 2002). Furthermore, it has been reported that excessive retinal glutamate excitotoxicity contributes to the upregulation of (VEGF) production in diabetic retinas, which is known to induce microcirculatory abnormalities such as disruption of the (BRB) or an increase in the vascular permeability in the advanced stage of DR (Cervantes-Villagrana et al., 2010; Kusari et al., 2010; Ishikawa, 2013). In addition, Kusari et al. (2010), demonstrated that the elevation of VEGF expression and BRB breakdown in STZ-induced diabetic rats is blocked by the NMDA receptor channel blocker and uncompetitive antagonist memantine (Kusari et al., 2010). This explains the relationship between the excitotoxicity and the induction of VEGF in the linking neurodegeneration with vascular impairment in DR. Furthermore, high levels of oxidative stress in diabetics have been proven to be associated with increased glutamate levels, which in turn directly contributes to DR (Narayanan and Shosha, 2019). Glutamate toxicity has been related to the inhibition of glutamate/cystine antiporter activity, which imports cystine to export glutamate outside the cell (Lewerenz et al., 2013). Cystine is required to synthesize the potent intracellular antioxidant glutathione (GSH). When Glu concentration is high outside the cell leads, it inhibits cystine uptake, depleting GSH synthesis in neuronal cells and inducing oxidative stress and apoptosis (Lewerenz et al., 2013). Several studies have also reported decreased GSH levels in diabetic retinal cells and increased oxidative stress (Al-Dosari et al., 2017; Ola et al., 2019).

### Arginine

L-Arginine is metabolized to nitric oxide (NO) and L-citrulline by NO synthases, and to urea and L-ornithine by arginase where the latter is further metabolized to form proline, polyamines, and glutamate. There is some evidence supporting the role of arginase in retinal inflammation, neurotoxicity, pathological angiogenesis, and retinal vascular dysfunction (Narayanan et al., 2013; Hou et al., 2021). Increased arginase activity, elevated expression of arginase, and decreased levels of L-arginine have been reported in plasma of diabetic animals and patients. Several studies have shown that increases in arginase activity are involved in diabetes and high glucose-induced dysfunction of the aorta, coronary and retinal arteries. Increases in arginase activity and arginase expression in neurovascular cells are associated with increased formation of superoxide, increased expression of inflammatory genes, and attachment of leukocytes to the retinal vessels (Elms et al., 2013; Narayanan et al., 2013; Patel et al., 2013; Fouda et al., 2020) High arginase activity leads to the uncoupling of nitric oxide synthase. Uncoupled NOS reacts with molecular oxygen to form superoxide, which will respond rapidly with any available NO to create peroxynitrite, a highly reactive inflammatory and toxic oxidant. Peroxynitrite causes cellular injury either through direct oxidative reactions with lipids, DNA, and proteins or indirect, radical-mediated mechanisms. In addition, inhibiting nitric oxide synthase (NOS) has been shown to prevent diabetes-induced vascular dysfunction implying that reactive nitrogen species play a crucial role in the DR pathology (Elms et al., 2013; Narayanan et al., 2013). Furthermore, *in vivo* experiments revealed that the long-term oral administration of L-citrulline offered protection against hyperglycemia-induced retinal vasodilator dysfunction by stimulates nitric oxide (NO) biosynthesis via the recycling of L-citrulline to L-arginine (Mori et al., 2015, 2021).

In addition, arginase catabolizes L-arginine to form proline, polyamines, and glutamate. The catabolic products of glutamate and polyamine oxidation have been linked to retinal ganglion cell death due to excessive activation of the excitotoxic NMDA receptors, which can induce more oxidative stress and DNA damage (Narayanan et al., 2013). Furthermore, polyamines have been implicated in neurovascular dysfunction in the retina. Polyamine production is further metabolized to spermine and spermidine (Narayanan et al., 2013; Narayanan and Shosha, 2019). Nicoletti et al. (2003), reported that the level of spermidine and spermine was increased in vitreous samples from patients with proliferative retinopathy, suggesting their involvement in retinopathy (Nicoletti et al., 2003). In addition, in the retinal arterioles, spermine inhibits potassium efflux via inwardly rectifying potassium channels, causing it to limit vasodilator responses and is enhanced in microvessels isolated from the diabetic retina.

### Branched Chain Amino Acid

BCAAs (valine, leucine, and isoleucine) are essential amino acids needed for protein synthesis, cells' energy needs, and signaling molecules. They are required for growth and development and act as nutrient signals and nitrogen donors for neurotransmitter synthesis and glutamate/glutamine cycling in the brain and retina (LaNoue et al., 2001; Conway and Hutson, 2016). Many reports have consistently demonstrated increased plasma BCAA levels related to obesity, insulin resistance, and diabetes in human studies and experimental rodents during the last decade (Bloomgarden, 2018). Besides, our lab and a few recent metabolomics investigations have shown significant changes in the levels of BCAA in the retina of diabetic animals and the vitreous of diabetic patients with proliferative diabetic retinopathy (Gowda et al., 2011; Bloomgarden, 2018; Ola et al., 2019). In addition, many neurological disease studies found that the build-up of BCAAs in the brain is neurotoxic to cells by increasing glutamate production, which induces excitotoxicity and neuronal death in the brain through mechanisms of NMDA receptor activation. In addition, the antiepileptic drug gabapentin, which is known to inhibit BCAT, an essential enzyme in the BCAA metabolism, has proved successful in reducing caspase-3 activity, a significant marker of oxidative stress, and reducing the increased levels of ROS in the diabetic retina (Ola et al., 2019). Thus, diabetes-induced altered BCAAs metabolism seems to be the potential in damaging neuroretina.

### Serine

Serine is a non-essential amino acid but essential in cellular homeostasis, proliferation, and differentiation (Sinha et al., 2020). Free serine is necessary for generating cysteine, glycine, and sphingolipids, which are critical in synthesizing porphyrins/purine nucleotides, glutathione, sphingomyelin formation, respectively (Sinha et al., 2020). Serine metabolism is central to maintaining redox/oxidative balance, ion flux, glutamate levels, and other support functions. Emerging evidence has demonstrated a correlation between serine deficiency and systemic diabetes (Holm and Buschard, 2019; Sinha et al., 2020). Reduced serine levels and increased D-serine have been implicated in the etiology of DR (Ozaki et al., 2018; Sinha et al., 2020). D-serine, the racemization of L-serine forms an enantiomer of L-serine by serine racemase (SRR) in Müller cells. D-serine has been found to have a high affinity for binding and activation of NMDA receptors in the retina (Sinha et al., 2020). Therefore, the elevated levels of D-serine contribute to the glutamate toxicity effect in the retinal environment and induce cell death in RGCs.

### Homocysteine

Homocysteine is a sulfur-containing non-proteinogenic amino acid, and its high levels are associated with various ocular complications, including secondary glaucoma optic atrophy, age-related macular degeneration (AMD), and DR (Ola et al., 2013). Homocysteine is biosynthesized from methionine by S-adenosyl-methionine synthetase in the presence of vitamin B12 and folate as cofactors. Homocysteine formed can either be remethylated back to L-methionine by methylenetetrahydrofolate reductase (MTHFR) and/or trans-sulfuration by cystathionine β-synthase (CBS) to form cysteine, an essential amino acid for the biosynthesis of glutathione (GSH) (Kowluru et al., 2020). Hyperhomocysteinemia in diabetic complications has been associated with impaired activities of CBS and MTHFR, along with deficiencies in folate and vitamin B12 (Kowluru et al., 2020). Naggar et al. (2002), demonstrate that hyperglycemia reduces the expression and activity of the folate transporter and decreases the folate level in the retina, which could have implications for the pathology of DR (Naggar et al., 2002). In addition, supplementation with folic acids and vitamin B is more effective in reducing homocysteine levels and maintaining homocysteine metabolism.

The elevated level of Hcy has a toxic effect on neuronal cells in both the brain and retina. A recent meta-analysis demonstrated an association between high Hcy levels and increased risk of diabetic retinopathy (Lei et al., 2018). In addition, hyperhomocysteinemia in DR has been associated with vitamin-B12 deficiency. *In vitro* and *in vivo* studies have also demonstrated vital roles for elevated homocysteine levels in apoptotic mechanisms leading to ganglion cell loss (Moore et al., 2001; Ganapathy et al., 2009). Several studies have shown that the neurotoxic effects of homocysteine are also associated with the activation of Glu receptors and cause excitotoxicity of retinal ganglion cells in the diabetic retina (Ola et al., 2013). A case study reported a high level of Hcy caused by methionine synthase deficiency demonstrated decreased rod function and RGC loss as determined by ERG and visual evoked potentials, which reflect homocysteine's cytotoxic impact on neurons of the visual pathway (Poloschek et al., 2005).

Additionally, more recently metabolomics approaches have been used by Sun and coworkers for plasma metabolic profiles in patients with DR to better understand the mechanism of this disease and the disease progression. Out of several differentially expressed metabolites associated with DR, a risk score based analysis identified pseudouridine to be strongly associated with the occurrence of DR. Furthermore, circulating plasma metabolites; glutamate, leucylleucine, and N-acetyltryptophan were found to be differentially expressed between patients with PDR and evaluated to be significantly related to PDR (Sun et al., 2021). The same group also identified arginine and carnitine metabolites which are altered in the plasma of DR patients. These metabolites can also be used as biomarkers in the disease progression (Sumarriva et al., 2019).

## Conclusion and Future Perspectives

Diabetic retinopathy is well recognized as a neurovascular disease. The pathophysiology of neurovascular damage is extremely complex, nevertheless, diabetes-induced metabolic disorders are considered the major initiating factors that damage both neuronal and vascular components of the retina. Besides diabetes-induced hyperglycemia, emerging evidence suggests a potential role of dyslipidemia and metabolic defects of amino acids that induce the early neurovascular retinal damage in the DR. However, the connections between diabetes-induced metabolic profiling changes and the exact pathways leading to the development of retinal pathology are still unknown. New tools are becoming available for metabolic profiling studies including specific metabolic enzyme inhibitors, antibodies, and animal genetic models to elucidate the metabolic defects. These strategies would contribute to creating a better understanding of the mechanism of diabetes-induced metabolic damage to allow the development of therapeutic options for the prevention and treatment of the neurodegenerative disease as in the case of DR.

## Data Availability Statement

The original contributions presented in the study are included in the article/supplementary files, further inquiries can be directed to the corresponding author/s.

## Author Contributions

DA designed the outline, wrote the first draft, and made figures. AM and AA reviewed and revised it. MO offered guidance, advice to design of the outline as well as wrote and revised, and edited the manuscript. All authors contributed to the article and approved the submitted version.

## Funding

This research was funded by King Abdul Aziz City for Science and Technology (KACST-NPST), grant number: 13-MED 1374.

## Conflict of Interest

The authors declare that the research was conducted in the absence of any commercial or financial relationships that could be construed as a potential conflict of interest.

## Publisher's Note

All claims expressed in this article are solely those of the authors and do not necessarily represent those of their affiliated organizations, or those of the publisher, the editors and the reviewers. Any product that may be evaluated in this article, or claim that may be made by its manufacturer, is not guaranteed or endorsed by the publisher.
